# Variation in the E2-binding domain of HPV 16 is associated with high-grade squamous intraepithelial lesions of the cervix

**DOI:** 10.1054/bjoc.2001.1695

**Published:** 2001-04

**Authors:** A Giannoudis, M van Duin, P J F Snijders, C S Herrington

**Affiliations:** Department of Pathology, University of Liverpool, Royal Liverpool University Hospital, Duncan Building, Daulby Street, Liverpool, L69 3GA, UK; Department of Pathology, University Hospital Vrije Universiteit, De Boelelaan 1117, Amsterdam, HV, 1081, The Netherlands

**Keywords:** cervix, squamous intraepithelial lesion, papillomavirus, HPV 16, variation

## Abstract

Human papillomaviruses (HPV) are strongly associated with cervical intraepithelial neoplasia (CIN) and invasive cancer mainly through the action of the E6 and E7 viral proteins, transcription of which is down-regulated by the E2 protein. To test the hypothesis that HPV 16 E2 variation is important in the development of high-grade squamous neoplasia of the cervix, we carried out a cross-sectional analysis of low-grade and high-grade squamous intraepithelial lesions (SILs) for specific mutations in the HPV 16 E2 gene and for E2 gene disruption in these regions. Isolates were also analysed for the HPV 16 350T-G variant. 22 of 178 low-grade SILs and 43 of 61 high-grade SILs examined, contained HPV 16. No relationship was found between the E6 350T-G variant, or the E2 hinge region 3410C-T variant, and lesion grade. However, disruption of the regions of E2 analysed was significantly more frequent in high-grade lesions, and there was a significant association between the 3684C-A variant in the E2 DNA binding domain and high-grade histology suggesting that this variant may be important in progression to high-grade intraepithelial disease. © 2001 Cancer Research Campaign http://www.bjcancer.com


*British Journal of Cancer* (2001) **84**(8), 1058–1063
© 2001 Cancer Research Campaign
doi: 10.1054/ bjoc.2001.1695, available online at http://www.idealibrary.com on IDEAL®

### Variation in the E2-binding domain of HPV 16 is associated with high-grade squamous intraepithelial lesions of the cervix

**A Giannoudis[1], M van Duin[2], PJF Snijders[2] and CS Herrington[1]**

[1]University of Liverpool, Department of Pathology, Royal Liverpool University Hospital, Duncan Building, Daulby Street, Liverpool, L69 3GA, UK;
[2]University Hospital Vrije Universiteit, Department of Pathology, De Boelelaan 1117, 1081 HV Amsterdam, The Netherlands

**Summary** Human papillomaviruses (HPV) are strongly associated with cervical intraepithelial neoplasia (CIN) and invasive cancer mainly through the action of the E6 and E7 viral proteins, transcription of which is down-regulated by the E2 protein. To test the hypothesis that HPV 16 E2 variation is important in the development of high-grade squamous neoplasia of the cervix, we carried out a cross-sectional analysis of low-grade and high-grade squamous intraepithelial lesions (SILs) for specific mutations in the HPV 16 E2 gene and for E2 gene disruption in these regions. Isolates were also analysed for the HPV 16 350T-G variant. 22 of 178 low-grade SILs and 43 of 61 high-grade SILs examined, contained HPV 16. No relationship was found between the E6 350T-G variant, or the E2 hinge region 3410C-T variant, and lesion grade. However, disruption of the regions of E2 analysed was significantly more frequent in high-grade lesions, and there was a significant association between the 3684C-A variant in the E2 DNA binding domain and high-grade histology suggesting that this variant may be important in progression to high-grade intraepithelial disease. © 2001 Cancer Research Campaign http://www.bjcancer.com

**Keywords**: cervix; squamous intraepithelial lesion; papillomavirus; HPV 16; variation

Human papillomavirus (HPV) infection is the main risk factor for the development of cervical squamous intraepithelial lesions (SILs) and carcinoma. To date, more than 80 different types of HPV have been identified and classified as low-risk (e.g. HPV 6, 11, 42, 43, 44) and high-risk (e.g. HPV16, 18, 31, 33, 35, 39, 45, 51, 52, 56, 58, 68) based on their association with SIL and cervical carcinoma. In particular, HPV 16 is found in only a small proportion of low-grade SILs but in a much higher proportion of high-grade SILs and invasive squamous carcinomas (Bosch et al, 1995).

HPVs are associated with cervical neoplasia through the action of two of their early (E) proteins, E6 and E7, transcription of which is regulated by the E2 viral protein through its binding to sites adjacent to the promoter responsible for this transcription (Howley et al, 1996; Kovelman et al, 1996). Consequently, increased expression of E2 results in repression of E6 and E7 expression and vice versa (Dowhanick et al, 1995; Howley et al, 1996). In addition, integration of the viral genome into host DNA, observed in the majority of cervical carcinomas and less commonly in pre-invasive lesions, often occurs in the region of the E1/E2 open reading frames (ORFs) and leads to deletion or disruption of E1 and/or E2 (Kalantari et al, 1998). Loss of E2 function as a consequence of integration results in up-regulation of E6/E7 gene transcription and overexpression of E6/E7 proteins leading to deregulated cell proliferation.

*Received 19 October 2000*
*Revised 2 January 2001*
*Accepted 11 January 2001*

*Correspondence to:* CS Herrington

E2 function can also potentially be altered by gene mutation/ variation. Genomic variation has been extensively studied for HPV 16-E6 in an attempt to identify naturally occurring variants that may exhibit altered biological functions (Stoppler et al, 1996; Zehbe and Tommasimo, 1999). Data examining the E2 gene are however scanty. In this study, we have investigated specific sequence variants from the E2 and E6 genes of HPV 16 in low- and high-grade SILs of the cervix to test the hypothesis that sequence variation is involved in disease progression. The variants analysed were chosen from the literature as they are in positions important for specific biological or immunological functions. From the hinge region, we analysed the 3410C-T (P219S), the 3516C-A (T254N) and the 3538A-C (S261-) variants present in antigenic domains (Gaulthier et al, 1991; Terry et al, 1997). From the DNA binding domain we analysed the 3684C-A (T310K) and the 3694T-A (T313-) variants (Veress et al, 1999). The first of these variants could be functionally important given that the DNA-binding activity of E2 is important for its function. The second allows distinction between the European variant present in Caski and SiHa cells (3684C-A, 3694prototype) and Asian-American variants (3684C-A and 3694T-A), which contain more widespread variation across the E2 gene (Casas et al, 1999). From the E6 gene we analysed the 350T-G variant which has been related to viral persistence and disease progression (Yamada et al, 1995; Londesborough et al, 1996; Zehbe and Tomassimo, 1999). A significant association was identified between the 3684C-A variant (T310K) in the DNA-binding domain of E2, but not the other variants analysed, and lesion grade. Our data suggest that the T310K variant may be linked to progression from low-to high-grade SILs, in lesions infected with HPV 16.

### MATERIALS AND METHODS

### Tissue specimens

Tissue specimens from 239 squamous intraepithelial lesions (SILs) were identified from the diagnostic files of the department of Pathology, Royal Liverpool University Hospital. The 178 low-grade SILs comprised condylomata (exophytic and flat) and cervical intraepithelial neoplasia (CIN) grade 1, the 61 high-grade SILs comprised CIN grade 3. All of the diagnoses were confirmed by CS Herrington. Paraffin-embedded archival tissues were used as sources of DNA.

### DNA extraction

Three 5 μm formalin-fixed, paraffin sections of cervical biopsy material were deparaffinized in xylene, washed with 96% ethanol, pelleted, air-dried and incubated overnight at 55˚C with 200 μg $ml^{-1}$ proteinase K (Boehringer Mannheim) in 200 mM Tris-HCl pH 8.0, 1 mM EDTA. The digest was heated to 95˚C for 20 min to arrest enzyme activity and centrifuged at 13 000 ***g*** for 5 min. The supernatant was used directly in the PCR reaction. A fragment of the p53 gene (307 bp) was amplified in each case using the primers p53+ and p53– to ensure the presence of amplifiable DNA (Giannoudis et al, 1999).

### HPV typing by PCR and hybridization

15 μl of the extracted DNA were used in a 50 μl PCR reaction. A 140–150 bp fragment from the HPV L1 gene was amplified using the consensus GP5+/6+ system (Jacobs et al, 1995). 10 μl of the PCR product were analysed on a 1.7% agarose gel stained with ethidium bromide. Dot-blot hybridization was carried out using a 5′ digoxigenin labelled oligonucleotide probe for HPV type 16 as previously described (Southern et al, 1997).

### PCR amplification of HPV 16-E2

15 μl of the extracted DNA were used in a 50 μl PCR reaction containing 50 mM KCl, 10 mM Tris-HCl (pH 8.3), 200 μM of each dNTP, 1.5 mM $MgCl_2$, 1 U of Taq DNA polymerase (Gibco) and 50 pmol of each primer. An initial denaturation step at 94˚C, 1 min and a final extension step at 72˚C, 5 min were performed for all the PCRs. The conditions of each PCR with the primers, their position in the E2 gene and the amplified product are summarized in Table 1A.

### Restriction fragment length polymorphism analysis

Variants in the E2 gene were identified by RFLP analysis. The restriction enzyme used for each sequence variant, their recognition sites and the fragments generated in wild-type and variant isolates are summarized in Table 1B.

### E2 sequencing

To confirm the results obtained from RFLP analysis we sequenced bidirectionally a number of representative samples of variants and wild-type isolates. All sequencing was performed in an ABI Prism™ 373 automated sequencer, using the same primers as for PCR.

**Table 1** (**A**) PCR conditions with the different primers and their position on the E2 gene. (**B**) The restriction enzymes used for RFLP analysis, their recognition sequences and the fragments generated

(A)

| E2 primers (5′–3′) | Positions | Product | Conditions |
|---|---|---|---|
| A1: GTAATAGTAACACTACACCCATA<br>A2: GGATGCAGTATCAAGATTTGT | 3597<br>3872 | 277 bp | Denaturation: 94˚C/1min<br>Annealling: 58˚C/1 min<br>Extension: 72˚C/1 min |
| B1: TAGCAGCAACGAAGTATCCTCTCC<br>B2: TGGATAGTCGTCTGTGTTTCTTCG | 3355<br>3471 | 116 bp | Denaturation: 94˚C/1 min<br>Annealling: 57˚C/1 min<br>Extension: 72˚C/1,5 min |
| C1: CGAAGAAACACAGACGACTATCCA<br>C2: TAAAGTATTAGCATCACCTT | 3448<br>3649 | 203 bp | Denaturation: 94˚C/1 min<br>Annealling: 50˚C/1 min<br>Extension: 72˚C/1,5 min |

(B)

| E2 mutation | Restriction enzyme | Recognition site | Restriction fragments | |
|---|---|---|---|---|
| | | | Wild-type | Mutant |
| 3684C-A | Rsa I (Promega) | 5′..GT/AC..3′ | 192+61+24 bp | 253+24 |
| 3694T-A | Pst I (Promega) | 5′..CTGCA/G..3′ | 177+100 bp | 277 bp |
| 3410C-T | Eci I (NEB) | 5′../TCCGCC..3′ | 116 bp | 61+55 bp |
| 3516C-A<br>3538A-C | Dde I (Promega) | 5′..C/TNAG..3′ | 115+69+19 bp | 116+87 bp<br>134+69 bp |

© 2001 Cancer Research Campaign

### Controls

Amplimers from CaSki cell DNA, which contains 3410C-T and 3684C-A variants, and the HPV 16 plasmid clone (prototype) were used as controls both in RFLP analysis and sequencing.

### PCR amplification of HPV16-E6 350T/G

HPV 16 E6 350T/G analysis was performed in 2 independent PCR reactions using the primers 350-1/350-3, specific for 350G and 350-2/350-3, specific for 350T, as previously described (Van Duin et al, 2000). The PCR products were analysed on 1.5% agarose gels. Southern blot hybridization was carried out using a [$\gamma$-$^{32}$P]ATP 3′-end labelled oligonucleotide probe at 55˚C (Snijders et al, 1998).

### Statistics

The relationships between the different variables were assessed using Fisher's exact test. A *P* value of less than 0.05 was considered statistically significant.

### RESULTS

### HPV 16 typing

Using the consensus primers GP5+/GP6+, HPV DNA was identified in all of the 239 SILs examined. HPV 16 DNA was identified by PCR and typing in 22/178 (12%) low-grade SILs and 43/61 (70%) high-grade SILs.

### Disruption of the E2 fragments

In all low-grade SILs amplification was achieved with all of the primer pairs used. In high-grade SILs failure of amplification was observed in 6/43 samples using the primers A1–A2, 4/43 using B1–B2 and 5/43 samples using C1–C2. No isolate showed failure of amplification of more than one fragment. This failure of amplification, despite amplification of an internal control with a larger product size, is consistent with disruption of the E2 gene. Disruption at these sites was significantly more frequent in high-grade lesions (15/43, 35%) than in low-grade lesions (0/22, 0%) (*P* = 0.0012, Fisher's exact test) (Table 2).

### E2 sequence variation

Sequencing analysis of representative isolates, and of the CaSki and HPV 16 plasmid products, gave the appropriate sequences. In the DNA binding domain of E2, the 3684C-A (T310K) variant (Figure 1A, lanes: 1–4) was identified in 13/38 (34%) high-grade SILs and 2/22 (9%) low-grade SILs by RFLP (*P* = 0.03, Fisher's exact test). The 3694T-A (T313-) (Figure 1A, lanes: 5–8) variant was identified in one low-grade SIL: this isolate also carried the 3410C-T, and the 3684C-A variants. None of the isolates from high-grade SILs contained this sequence variant.

RFLP analysis of the amplified DNA samples revealed that the 3410C-T variant (P219S) within the hinge region (Figure 1B, lanes: 4, 5), was present in 27/37 (73%) amplifiable high-grade SILs and 12/21 (57%) low-grade SILs (*P* = 0.25, Fisher's exact test). Neither the 3516C-A (T254N) nor the 3538A-C (S261-) (Figure 1B, lanes: 1–3) sequence variant was identified in the 39 amplifiable high-grade SILs or the 21 amplifiable low-grade SILs. In one of the 22 low-grade SILs insufficient DNA was available to perform analysis of 3410C-T, 3516C-A, or 3538A-C variants.

25 of 43 (58%) high-grade SILs contained isolates with either E2 disruption in the regions analysed or the 3684C-A (T310K) variant compared with only 2 of 22 (9%) low-grade SILs (*P* = 0.0001, Fisher's exact test).

### E6 350T-G analysis

From the 36 high-grade SILs that gave amplification signal for the 350T-G, 21 (58%) isolates were prototype, 14 (39%) contained the 350G variant and 1 (3%) contained both 350T and 350G. 7 samples failed to amplify: this could be due to the incorporation of an artificial mismatch at the second 3′ nucleotide position of the forward primers specific for T or G in order to increase specificity but failing to achieve the most optimal sensitivity (Snijders et al, 1998). An alternative, albeit unlikely, possibility is that these samples may contain a base different from T or G.

9 of 20 (45%) low-grade SILs contained the E6 350T prototype, 7 (35%) contained the 350G variant and 4 (20%) contained both 350T and 350G. In 2 of the 22 samples insufficient material was available to perform this analysis.

### HPV 16 E6 and E2 variants

Sequence variation in E2 in combination with the E6 350T-G variant was identified in 9/14 (64%) and 11/26 (42%) low- and high-grade SILs respectively (Table 2). The E6 350T-G variants were compared to both E2 3410C-T and 3684T-A variants in isolates in which E2 was not disrupted within the relevant region, and E6 350T-G analysis showed the presence of a single variant type. The E6 350G variant was present in none of the 12 isolates with 3410C prototype but was identified in 19/36 isolates with variant 3410T (*P* = 0.0013, Fisher's exact test). The E6 350G variant was identified in none of the 13 isolates with 3684A variant but was present in 18/34 isolates with 3684C prototype (*P* = 0.0006, Fisher's exact test). Thus, the 350G variant segregates with the 3684C prototype and the 350T prototype segregates with the 3684A variant.

### DISCUSSION

In this study we have analysed specific E2 sequence variants in HPV 16 isolates from low-and high-grade SILs of the cervix. Our data indicate that (i) the E2 T310K variant segregates with lesion grade independently of other HPV 16 E6 and E2 variants and, (ii) the HPV 16 E2 gene is frequently disrupted in the regions analysed in high-grade SILs but not in low-grade SILs.

HPV 16 infection is observed in 10–15% of low-grade SILs and 60–90% of high-grade SILs and cervical cancers. Risk factors in addition to infection have been identified but their exact role in the development of high-grade lesions and invasive cervical carcinoma are not fully elucidated (Southern and Herrington, 1998). However, expression of the early viral proteins E6, E7 and E2 play an important role in the disruption of cell cycle control and are likely therefore to be pivotal in the acquisition by infected cells of subsequent genetic abnormalities. In particular, as E2 negatively regulates transcription of the E6 and E7 genes, loss of function of E2, through either disruption or mutation, leads to up-regulation of

© 2001 Cancer Research Campaign

**Table 2** Summary of results

| No | Diagnosis | 3410C-T | 3516C-A | E2 3538A-C | 3684C-A | 3694T-A | E6 350T-G |
|---|---|---|---|---|---|---|---|
| 1 (S) | CIN 1 | – | – | – | – | – | – |
| 2 (S) | CIN 1 | T | – | – | A | – | – |
| 3 | CIN 1 | – | – | – | – | – | – |
| 4 (S) | CIN 1 | T | – | – | – | – | G |
| 5 | CIN 1 | – | – | – | – | – | – |
| 6 (S) | CIN 1 | – | – | – | – | – | – |
| 7 (S) | CIN 1 | T | – | – | – | – | G |
| 8 (S) | CIN 1 | T | – | – | – | – | G |
| 9 (S) | CIN 1 | T | – | – | – | – | G |
| 10 (S) | CIN 1 | T | – | – | – | – | – |
| 11 | CIN 1 | – | – | – | – | – | – |
| 12 (S) | CIN 1 | T | – | – | – | – | –/G |
| 13 (S) | CIN 1 | T | – | – | – | – | G |
| 14 (S) | CIN 1 | T | – | – | – | – | –/G |
| 15 (S) | CIN 1 | T | – | – | – | – | G |
| 16 | CIN 1 | – | – | – | – | – | – |
| 17 (S) | CIN 1 | T | – | – | – | – | G |
| 18 | CIN 1 | – | – | – | – | – | –/G |
| 19 | CIN 1 | – | – | – | – | – | INS |
| 20 | CIN 1 | T | A | – | A | A | – |
| 21 | CIN 1 | – | – | – | – | – | –/G |
| 22 | CIN 1 | INS | INS | INS | – | – | INS |
| 1 (S) | CIN 3 | T | – | – | – | – | – |
| 2 | CIN 3 | NA | – | – | A | – | NA |
| 3 (S) | CIN 3 | T | – | – | A | – | – |
| 4 (S) | CIN 3 | – | – | – | – | – | – |
| 5 (S) | CIN 3 | T | – | – | A | – | – |
| 6 | CIN 3 | NA | – | – | – | – | G |
| 7 (S) | CIN 3 | T | – | – | – | – | G |
| 8 | CIN 3 | NA | – | – | – | – | G |
| 9 | CIN 3 | T | – | – | NA | NA | NA |
| 10 (S) | CIN 3 | T | – | – | – | – | G |
| 11 (S) | CIN 3 | – | – | – | – | – | NA |
| 12 | CIN 3 | T | NA | NA | A | – | – |
| 13 | CIN 3 | NA | – | – | – | – | G |
| 14 (S) | CIN 3 | T | – | – | A | – | – |
| 15 (S) | CIN 3 | T | – | – | A | – | – |
| 16 | CIN 3 | T | – | – | A | – | NA |
| 17 | CIN 3 | – | NA | NA | – | – | NA |
| 18 (S) | CIN 3 | T | – | – | A | – | – |
| 19 (S) | CIN 3 | T | – | – | – | – | G |
| 20 | CIN 3 | – | – | – | – | – | –/G |
| 21 | CIN 3 | NA | – | – | – | – | NA |
| 22 (S) | CIN 3 | – | – | – | – | – | – |
| 23 | CIN 3 | T | NA | NA | A | – | – |
| 24 (S) | CIN 3 | T | – | – | – | – | – |
| 25 (S) | CIN 3 | T | – | – | A | – | – |
| 26 (S) | CIN 3 | T | – | – | – | – | G |
| 27 | CIN 3 | – | – | – | – | – | – |
| 28 | CIN 3 | T | – | – | NA | NA | G |
| 29 (S) | CIN 3 | T | – | – | A | – | – |
| 30 (S) | CIN 3 | T | – | – | – | – | – |
| 31 | CIN 3 | – | – | – | – | – | – |
| 32 (S) | CIN 3 | T | – | – | – | – | G |
| 33 (S) | CIN 3 | T | – | – | – | – | G |
| 34 | CIN 3 | T | – | – | NA | NA | – |
| 35 (S) | CIN 3 | T | – | – | – | – | G |
| 36 | CIN 3 | – | – | – | – | – | NA |
| 37 (S) | CIN 3 | – | – | – | A | – | – |
| 38 | CIN 3 | NA | – | – | – | – | – |
| 39 (S) | CIN 3 | T | – | – | A | – | – |
| 40 | CIN 3 | – | – | – | – | – | – |
| 41 | CIN 3 | T | NA | NA | – | – | G |
| 42 | CIN 3 | T | – | – | NA | NA | G |
| 43 | CIN 3 | T | – | – | NA | NA | G |

NA = Non-amplifiable; INS = insufficient material; (S) = sequenced.

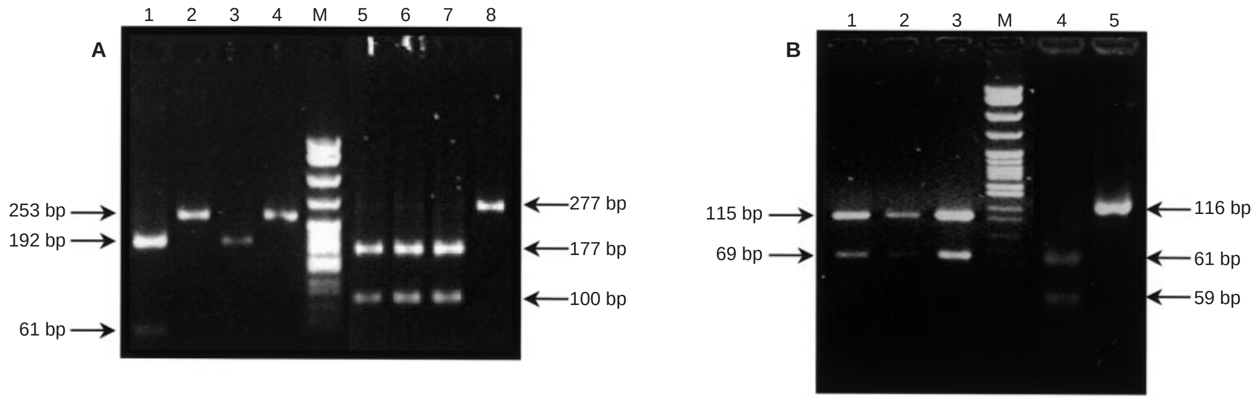

**Figure 1** Restriction fragment length polymorphism analysis. The plasmid pBR322 digested with Msp I was used as molecular size marker (M). (**A**) Lanes 1–4: PCR products digested with Rsa I. Wild type E2 (lanes 1, 3) giving 192, 61 and 24 bp (not visible) fragments. T310K variant (lanes 2, 4) giving 253 and 24 bp fragments. Lanes 5–8: PCR products digested with Pst I. Wild-type E2 (lanes 5–7) giving 177 and 100 bp fragments. T313- (lane 8) loses the restriction site, giving full length PCR product. (**B**) Lanes 1–3: PCR products digested with Dde I showing wild-type E2 giving 115, 69 and 19 bp (not visible) fragments. Lanes 4–5: PCR products digested with Eci I. Wild-type E2 (lane 5) giving 116 bp fragment. P219S variant (lane 4) giving 61 and 55 bp fragments the HPV early promoter and increased expression of the E6/E7 proteins (Dowhanick et al, 1995; Howley, 1996).

We identified disruption of one or more of the fragments of the E2 gene examined in 35% of high-grade SILs but in none of the low-grade SILs. These data are consistent with the fact that viral integration is found more frequently in high-grade SILs and invasive carcinomas than in low-grade SILs. However, E2 disruption is not a prerequisite for the development of invasive disease. For example, intact E2 genes have been identified in invasive lesions where both episomal and integrated viral sequences are present. Moreover, the presence of an intact E2 gene in some lesions is consistent with the frequent detection of E2 antibodies in patients with high-grade SIL and invasive carcinoma (Dillner et al, 1995). In lesions containing intact E2 genes and expressing E2 protein, DNA sequence variation with consequent alteration in protein structure and possibly function may influence both the viral life cycle and neoplastic transformation.

In our study, the 3410C-T (P219S) variant, which is in the hinge region and is close to an antigenic domain (Gaulthier et al, 1991), was common but not related to lesion grade. Neither the 3516C-A (T254N) nor the 3538A-C (S261-) variants, both of which are present within another described antigenic domain (HPV 16 amino-acids 226–275) (Terry et al, 1997) and are within the sequence of the E4 gene, were identified in the lesions examined. 2 variants in the DNA-binding domain of the E2 gene were also examined. Variants in this domain could be biologically important given that the DNA binding activity of E2 is important for its function. The 3694T-A (T313-) was identified in only one low-grade SIL that was also carrying the 3410C-T, and the 3684C-A variants. The 3684C-A (T310K) variant was present in significantly more isolates from high-grade SILs than in isolates from low-grade SILs. The T310K substitution is immediately adjacent to the DNA-binding helix and comparison of prototype and variant sequences with the crystal structure of the BPV 1 DNA-binding domain and the NMR structure of the HPV 31 DNA-binding domain suggests that it may alter the conformation of the E2 protein. Although a functional study using the T310K variant revealed that its transactivation properties remain unaltered by comparison with the prototype (Veress et al, 1999), our data suggest that this substitution may be biologically relevant in vivo. One possible explanation for the differences is that the interaction between cellular transcription factors, the E2 protein and the viral promoter/enhancer region may be altered by the T310K variant. This was not addressed in the published in vitro functional study (Veress et al, 1999).

Finally, we examined the 350T-G (L83V) variant in the HPV 16 E6 gene. This sequence variation has been linked with viral persistence and lesion grade (Londesborough et al, 1996) but additional studies, conducted in different laboratories, found no association between its prevalence and lesion grade (Zehbe and Tomassimo, 1999). It has been suggested that the oncogenicity of this specific E6 variant may vary geographically, possibly due to differences in the HLA distribution. In our study, the 3684A variant segregated with the 350T prototype, not the 350G variant. Moreover, there was no significant difference between the prevalence of the E6 350G variant in low-and high-grade SILs, in agreement with previous studies (Zehbe and Tomassimo, 1999), demonstrating that this segregation was not responsible for the observed relationship between the 3684C-A variant and CIN 3. It is, however, difficult to exclude the possibility that other variants, which may co-segregate with this E2 variant, might be related to the observed clinical effect. The identification of both E6 350T and 350G in some lesions is consistent with a recent study in which the same phenomenon was reported (Emeny et al, 1999). It is of note that dual infection was present in a greater number of low-grade SILs than high-grade SILs. Although the number of lesions analysed is small, this observation is consistent with the decrease in prevalence of infection with multiple HPV types with increasing severity of cervical neoplasia (Chang et al, 1997). The biological significance of infection with multiple HPV variants remains to be determined.

In summary, no relationship was found between the E6 350T-G variant, or the E2 hinge region 3410C-T variant, and lesion grade. However, disruption of the regions of E2 analysed was significantly more frequent in high-grade lesions, and there was a significant association between the 3684C-A (T310K) variant in the E2 DNA-binding domain and high-grade histology. In addition, the 350T-G variant segregated with the 3410C-T but not with the 3684C-A variant indicating that the biological effect of 3684C-A is not a surrogate marker of 350T-G. These findings suggest that infection with HPV 16 variants containing the 3684C-A variant may be important in progression to high-grade intraepithelial disease.

 *© 2001 Cancer Research Campaign*

### ACKNOWLEDGEMENTS

We acknowledge grant VU 96–1156 of the Dutch Cancer Society and the University of Liverpool for funding this project.

### REFERENCES

Bosch FX, Manos MM, Munoz N, Sherman M, Jansen AM, Peto J, Schiffman MH, Moreno V, Kurman R, Shah KV and the International Biological Study of Cervical Cancer Study Group (1995) Prevalence of human papillomavirus in cervical cancer: a worldwide perspective. *J Natl Cancer Inst* **87**: 796–802

Casas L, Galvan SC, Ordonez RM, Lopez N, Guido M and Berumen J (1999) Asian-American variants of human papillomavirus type 16 have extensive mutations in the E2 gene and are highly amplified in cervical carcinomas. *Int J Cancer* **83**: 449–455

Chang DY, Chen RJ, Lee SC and Huang SC (1997) Prevalence of single and multiple infection with human papillomavirus in various grades of cervical neoplasia. *J Med Microbiol* **46**: 54–60

Dillner J, Zellbi A, Avall-Lundqvist E, Heino P, Eklund C, Pettersson CA, Forslund O, Hannsson BG, Graddien M and Bistoletti P (1995) Association of serum antibodies against defined epitopes of human papillomavirus L1, E2 and E7 antigens and of HPV DNA in incident cervical cancer. *Cancer Detect Prev* **19**: 381–393

Dowhanick JJ, McBride AA and Howley PM (1995) Suppression of cellular proliferation by the papillomavirus E2 protein. *J Virol* **69**: 7791–7799

Emeny RT, Herron JR, Xi LF, Koutsky LA, Kiviat NB and Wheeler CM (1999) Comparison of variant specific hybridisation and single-strand conformational polymorphism methods for detection of mixed human papillomavirus type 16 variant infections. *J Clin Microbiol* **37**: 3627–3633

Gaulthier J-M, Dillner J and Yanin M (1991) Structural analysis of the human papillomavirus type 16 E2 transactivator with antipeptide antibodies reveals a high mobility region linking the transactivation and the DNA binding domains. *Nucleic Acids Res* **19**: 7073–7079

Giannoudis A, Graham DA, Southern SA and Herrington CS (1999) The p53 codon 72 Arg/Pro polymorphism is not related to HPV type or lesion grade in low and high grade squamous intraepithelial lesions and invasive squamous carcinoma of the cervix. *Int J Cancer* **83**: 66–69

Howley PM (1996) Papillomaviridae: the viruses and their replication. In: *Fundamental Virology* (3rd ed), Fields BN, Knipe DM, Howley PM (eds). Lippincott-Raven Publishers: Philadelphia

Jacobs MV, de Roda Husman AM, van den Brule AJC, Snijders PJF, Meijer CJLM, and Walboomers JMM (1995) Group-specific differentiation between high and low risk human papillomavirus genotypes by general primer mediated PCR and two cocktails of oligonucleotide probes. *J Clin Microbiol* **33**: 901–905

Kalantari M, Karlsen F, Kristensen G, Holm R, Hagmar B and Johansson B (1998) Disruption of the E1 and E2 reading frames of HPV 16 in cervical carcinoma is associated with poor prognosis. *Int J Gynaecol Pathol* **17**: 146–153

Kovelman R, Bilter GK, Glezer E, Tsou AY and Barbosa MS (1996) Enhanced transcriptional activation by E2 proteins from the oncogenic human papillomaviruses. *J Virol* **70**: 7549–7560

Londesborough P, Ho L, Terry G, Cuzick J, Wheeler C and Singer A (1996) Human papillomavirus genotype as a predictor of persistence and development of histological grade lesions in women with minor cervical abnormalities. *Int J Cancer* **69**: 364–368

Snijders PJF, Van Duin M, Walboomers JMM, Steenbergen RDM, Risse EKJ, Helmerhorst TJM, Verheijen RHM and Meijer CJLM (1998) Telomerase activity exclusively in cervical carcinomas and a subset of cervical intraepithelial neoplasia grade III lesions: strong association with elevated mRNA levels of its catalytic subunit and high-risk human papillomavirus DNA. *Cancer Res* **58**: 3812–3818

Southern SA and Herrington CS (1998) Molecular events in uterine cervical cancer. *Sex Transm Inf* **74**: 101–109

Southern SA, Evans MF and Herrington CS (1997) Basal cell tetrasomy in low grade cervical squamous intraepithelial lesions infected with high risk human papillomaviruses. *Cancer Res* **57**: 4210–4213

Stöppler MC, Ching K, Stöppler H, Clancy K, Schlegel R and Icenogle J (1996) Natural variants of the human papillomavirus type 16 E6 protein differ in their abilities to alter keratinocyte differentiation and to induce p53 degradation. *J Virol* **70**: 6987–6993

Terry G, Ho L and Cuzick J (1997) Analysis of E2 amino acid variants of human papillomavirus types 16 and 18 and their associations with lesion grade and HLA/DR type. *Int J Cancer* **73**: 651–655

Van Duin M, Snijders PJF, Vossen MTM, Klaassen E, Voorhorst F, Verheijen RHM, Helmerhorst TJM, Meijer CJLM and Walboomers JMM (2000) Analysis of human papillomavirus type 16 E6 variants in relation to p53 codon 72 polymorphism genotypes in cervical carcinogenesis. *J Gen Virol* **81**: 317–325

Veress G, Szarka K, Dong X-P, Gergely L and Pfister H (1999) Functional significance of sequence variation in the E2 gene and the long control region of human papillomavirus type 16. *J Gen Virol* **80**: 1035–1043

Yamada T, Wheeler CM, Halpern AL, Stewart A-CM, Hildesheim A and Jenison SA (1995) Human papillomavirus type 16 variant lineages in United States populations characterised by nucleotide sequence analysis of the E6, L2 and L1 coding segments. *J Virol* **69**: 7743–7753

Zehbe I and Tomassimo M (1999) The biological significance of human papillomavirus type 16 variants and the development of cervical neoplasia. *Papillomavirus Rep* **10**: 105–116

© 2001 Cancer Research Campaign 
